# Characteristics and hotspots of the 50 most cited articles in the field of pre-psoas oblique lumbar interbody fusion

**DOI:** 10.3389/fsurg.2022.1004839

**Published:** 2022-10-12

**Authors:** Guang-Xun Lin, Chien-Min Chen, Shang-Wun Jhang, Ming-Tao Zhu, Pengfei Lyu, Bao-Shan Hu

**Affiliations:** ^1^ The School of Clinical Medicine, The Third Clinical Medical College, Fujian Medical University, China; ^2^ Department of Orthopedics, The First Affiliated Hospital of Xiamen University, School of Medicine, Xiamen University, Xiamen, China; ^3^ Division of Neurosurgery, Department of Surgery, Changhua Christian Hospital, Changhua, Taiwan; ^4^ Department of Leisure Industry Management, National Chin-Yi University of Technology, Taichung, Taiwan; ^5^ College of Nursing and Health Sciences, Dayeh University, Taiwan; ^6^ Department of Neurosurgery, The First Affiliated Hospital of Xiamen University, School of Medicine, Xiamen University, Xiamen, China; ^7^ Department of Breast Surgery, The First Affiliated Hospital of Hainan Medical University, Haikou, China

**Keywords:** anterior to psoas, citation analysis, most cited, oblique lumbar interbody fusion, prepsoas, OLIF

## Abstract

**Purpose:**

In the past decade, the field of pre-psoas oblique lumbar interbody fusion (OLIF) has developed rapidly, and with it, the literature on OLIF has grown considerably. This study was designed to analyze the top 50 articles in terms of the number of citations through bibliometric research to demonstrate the research characteristics and hotspots of OLIF.

**Method:**

Searching the Web of Science database yielded the 50 most cited publications in the OLIF field as of July 10, 2022. The publications were ranked according to the number of citations. The following sources were evaluated: the year of publications, the number of citations, authors, countries, institutions, journals, research topics, and keyword hotspots.

**Results:**

The most productive period was from 2017 to 2020, with 41 articles. The number of citations varied from 10 to 140, with an average of 35.52, and 1,776 citations were found. *World Neurosurgery* published the most articles (12), China produced the most articles (16), and the Catholic University of Korea produced the most studies (6). The corresponding author who produced the most articles was J.S. Kim (5), and the first author who produced the most publications was S. Orita (3). The main research topics were anatomical morphology, surgical techniques, indications, outcomes, and complications. The top 10 most cited keywords were “complications,” “decompression,” “spine,” “surgery,” “outcomes,” “transpsoas approach,” “spondylolisthesis,” “anterior,” “disease,” and “injury.”

**Conclusions:**

Certain articles can be distinguished from others using citation analysis as an accurate representation of their impact due to their long-term effectiveness and peer recognition. With these publications, researchers are provided with research priorities and hotspots through influential literature in the field of OLIF.

## Introduction

The anterior retroperitoneal approach, which was initially introduced by Mayer in 1997 and evolved through time, was termed oblique lateral lumbar interbody fusion (OLIF) by Silvestre et al. (1) in 2012 and has since become a popular and commonly used approach of lumbar interbody fusion (2). The OLIF procedure is distinguished by the fact that it does not require access to the abdominal cavity or incision of the psoas major muscle, thereby, preserving normal anatomy and allowing the placement of large interbody cages to fully restore disk height, achieve indirect decompression, and correct imbalances (3–5). Compared with the typical anterior approach technique, OLIF does not require extensive dissection or traction of the peritoneum, retroperitoneal arteries, and nerves, lowering the risk of vascular, visceral, and nerve injuries (6). The OLIF procedure, as opposed to direct/extreme lateral lumbar interbody fusion (D/XLIF), does not require crossing the lumbar major muscle, protecting it and avoiding the lumbar plexus nerve, resulting in a significantly lower incidence of lumbar plexus nerve injury and eliminating the need for intraoperative nerve monitoring (7, 8). Furthermore, compared with the posterior/transforaminal approach, OLIF does not destroy the muscles, ligament complexes, and bony structures of the posterior lumbar spine, which is more conducive to preserving the stability of the posterior lumbar column and generally does not cause damage to the spinal cord and nerve roots (9, 10). Therefore, annually, numerous experts and researchers endeavor to provide new insights into OLIF, and numerous articles on OLIF are published, proving its safety and effectiveness in the form of case reports, surgical technique descriptions, reviews, and clinical studies.

Citation analysis involves ranking articles based on the number of citations they receive, evaluating them and identifying influential studies in the field, and further applying a bibliometric analysis to these studies (11). While citation analysis studies remain somewhat controversial, proponents point to this method as an objective way in which the importance of an article or journal can be determined.

Citation analysis has been adopted in numerous medical fields to determine influential publications in their respective fields (12–14). However, so far, no citation analysis studies have been conducted focusing on OLIF. Given this situation, this study was designed to analyze the top 50 influential articles through citation analysis to visually present the research characteristics and hotspots of OLIF.

## Method

The data for this study were obtained from the Web of Science (WoS) core collection on July 10, 2022. The WoS is a critical database for worldwide access to academic content that is commonly used in citation analysis or bibliometric research (15–17). Furthermore, we used PubMed to identify supplementary data connected to the research.

The following search keywords were used: ((((TS = (oblique)) or TS = (anterior to psoas)) or TS = (anterior retroperitoneal)) or TS = (prepsoas)) and TS = (interbody fusion). From January 1982 to July 10, 2022, all English articles were limited to OLIF. Only original articles and reviews were included. Two independent reviewers confirmed their relevance to the OLIF publications according to their titles and abstracts. Any disagreements were resolved through discussion or by consulting a third reviewer until consensus was reached.

The top 50 OLIF-related articles with the most citations were obtained and reviewed. The title, author names, journal, year of publication, number of citations, and citations per year were documented. The region and institution of each article's author/s were recorded. If an article has more than one region or institution, the region and institution of the last corresponding author are recorded. The keywords were further visualized using the package bibliometrix through RStudio and VOSviewer (18).

## Results

**Figure 1:**
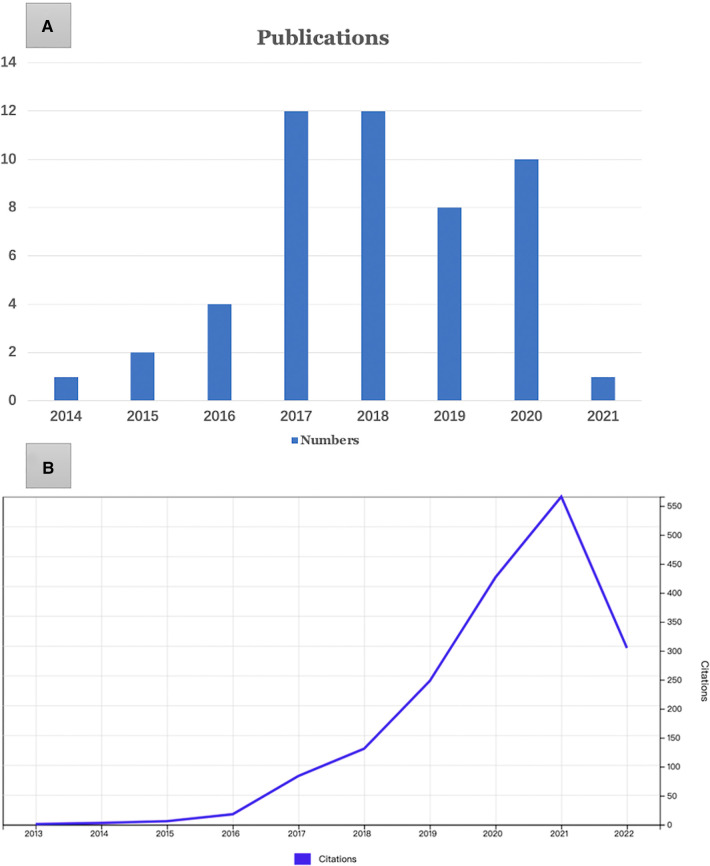
Annual number of publications (**A**) and citations (**B**).

Initially, 629 papers were searched, and after careful screening, 236 of them were related to OLIF, and finally the 50 most cited OLIF-related publications were identified. All 50 most cited articles were published between 2014 and 2021 (Figure 1). The most productive period was from 2017 to 2020, with 41 articles. Of these articles, the oldest was published by T.T. Davis et al. (19), and the most recent was published by B. Meng et al. (20). The number of citations varied from 10 to 140, with an average of 35.52, and 1,776 citations were found. Among them, four articles had more than 100 citations. Among these publications, the most cited paper was the study by S. Fujibayashi et al. (21), entitled “Effect of Indirect Neural Decompression Through Oblique Lateral Interbody Fusion for Degenerative Lumbar Disease.” Table 1 shows the 50 most cited papers based on the number of citations to better present the details to the investigators.

**Table 1:** Countries of the 50 most cited articles on pre-psoas oblique lumbar interbody fusion.

Rank	Country	Number	%
1	China	16	32
2	United States	14	28
3	South Korea	9	18
4	Japan	5	10
5	Australia	3	6
6	Italy	1	2
7	Canada	1	2
8	France	1	2

The 50 most cited articles were published in 22 journals (Table 2), with *World Neurosurgery* contributing the most publications (*n* = 12), followed by *European Spine Journal* (*n* = 5), *Spine* (*n* = 5), and *Orthopaedic Surgery* (*n* = 4).

**Table 2:** Contributing institution of the 50 most cited articles on pre-psoas oblique lumbar interbody fusion.

Rank	Institution	Country	Number	%
1	Catholic University of Korea	South Korea	6	12
2	Chiba University	Japan	4	8
3	Beijing Jishuitan Hospital	China	3	6
4	University of New South Wales	Australia	3	6
5	Anhui University	China	2	4
6	Army Medical University	China	2	4
7	Zhejiang University	China	2	4
8	Source Healthcare	United States	2	4
9	University of California System	United States	2	4

Eight countries contributed to the 50 most cited papers in the OLIF field (Table 3). China was the primary contributor (*n* = 16), followed by the United States (*n* = 14), South Korea (*n* = 9), Japan (*n* = 5), Australia (*n* = 3), and France, Italy, and Australia (*n* = 1 for each). No shortage of multinational collaborative papers was observed.

**Table 3:** Journal distribution of the 50 top cited articles on pre-psoas oblique lumbar interbody fusion.

Journal	Number	%
World Neurosurgery	12	24
European Spine Journal	5	10
Spine	5	10
Orthopaedic Surgery	4	8
Annals of Translational Medicine	2	4
BMC Musculoskeletal Disorders	2	4
Global Spine Journal	2	4
Journal of Neurosurgery Spine	2	4
Journal of Orthopaedic Surgery and Research	2	4
Neurosurgical Focus	2	4
Orthopaedics Traumatology Surgery Research	2	4
Clinical Spine Surgery	1	2
Clinical Orthopaedics and Related Research	1	2
Current Reviews in Musculoskeletal Medicine	1	2
Journal of Clinical Neuroscience	1	2
Journal of Comparative Effectiveness Research	1	2
Medicine	1	2
Neurosurgical Review	1	2
PloS One	1	2
Spine Journal	1	2
Yonsei Medical Journal	1	2

Table 4 shows the institutions with >2 publications among the 50 most cited publications in the field of OLIF. The Catholic University of Korea (South Korea) produced the most publications (*n* = 6), followed by Chiba University (Japan) (*n* = 4), Beijing Jishuitan Hospital (*n* = 3), and University of New South Wales (*n* = 3).

**Table 4:** Authors with multiple publications in the field of pre-psoas oblique lumbar interbody fusion.

Description	Author	Number	Affiliation
Most produced corresponding author	J.S. Kim	5	Department of Neurosurgery, Seoul St. Mary's Hospital, The Catholic University of Korea, Seoul, South Korea.
	R.J. Mobbs	3	NeuroSpine Clinic, Prince of Wales Private Hospital, University of New South Wales, Sydney, Australia
	W. Tian	2	Department of Neurosurgery, University of California San Francisco, San Francisco, CA, United States.
	S. Ohtori	2	Department of Orthopaedic Surgery, Graduate School of Medicine, Chiba University, Japan
	S. Orita	2	Department of Orthopaedic Surgery, Graduate School of Medicine, Chiba University, Japan
	C.L. Shen	2	Department of Orthopedics and Spine Surgery, The First Affiliated Hospital of Anhui Medical University, Anhui, China.
Most produced first author	S. Orita	3	Department of Orthopaedic Surgery, Kyoto University, Graduate School of Medicine, Kyoto, Japan.
	H.M. Li	2	Department of Orthopedics and Spine Surgery, The First Affiliated Hospital of Anhui Medical University, Anhui, China.
	D.H. Heo	2	Department of Neurosurgery, Leon Wiltse Memorial Hospital, Suwon, South Korea.
	J.X. Li	2	NeuroSpine Clinic, Prince of Wales Private Hospital, University of New South Wales, Sydney, Australia
Most produced co-author	J.S. Kim	6	Department of Neurosurgery, Seoul St. Mary's Hospital, The Catholic University of Korea, Seoul, South Korea.

Moreover, 272 authors contributed to the 50 most cited articles. Table 5 presents the first, last, and co-authors of the most cited articles. The corresponding author who produced the most publications was J.S. Kim (South Korea) (*n* = 5), and the first author who produced the most publications was S. Orita (Japan) (*n* = 3).

**Table 5:** Top 50 cited articles in the field of pre-psoas oblique lumbar interbody fusion.

Rank	Title	Author	Journal	Year	Total citations	Levels of evidence
1	Effect of Indirect Neural Decompression Through Oblique Lateral Interbody Fusion for Degenerative Lumbar Disease (21)	S. Fujibayashi et al.	Spine	2015	140	IV
2	Technical description of oblique lateral interbody fusion at L1-L5 (OLIF25) and at L5-S1 (OLIF51) and evaluation of complication and fusion rates (24)	K.R. Woods et al.	Spine Journal	2017	113	IV
3	Perioperative Complications in 155 Patients Who Underwent Oblique Lateral Interbody Fusion Surgery Perspectives and Indications from a Retrospective, Multicenter Survey (29)	K. Abe et al.	Spine	2017	104	IV
4	Radiographic evaluation of indirect decompression of mini-open anterior retroperitoneal lumbar interbody fusion: oblique lateral interbody fusion for degenerated lumbar spondylolisthesis (42)	J. Sato et al.	European Spine Journal	2017	102	IV
5	Minimally invasive anterior, lateral, and oblique lumbar interbody fusion: a literature review (43)	D.S. Xu et al.	Annals of Translational Medicine	2018	90	V
6	Retroperitoneal oblique corridor to the L2-S1 intervertebral discs in the lateral position: an anatomic study (19)	T.T. Davis et al.	Journal of Neurosurgery-Spine	2014	87	V
7	Mini-Open Anterior Retroperitoneal Lumbar Interbody Fusion: Oblique Lateral Interbody Fusion for Lumbar Spinal Degeneration Disease (44)	S. Ohtori et al.	Yonsei Medical Journal	2015	81	IV
8	The Oblique Anterolateral Approach to the Lumbar Spine Provides Access to the Lumbar Spine with Few Early Complications (45)	C. Mehren et al.	Clinical Orthopaedics and Related Research	2016	75	IV
9	Oblique Lumbar Interbody Fusion: Technical Aspects, Operative Outcomes, and Complications (25)	J.X. Li et al.	World Neurosurgery	2017	73	I
10	Review of early clinical results and complications associated with oblique lumbar interbody fusion (OLIF) (46)	K. Phan et al.	Journal of Clinical Neuroscience	2016	64	I
11	Complications and Prevention Strategies of Oblique Lateral Interbody Fusion Technique (47)	Z. Zeng et al.	Orthopaedic Surgery	2018	53	IV
12	MIS Single-position Lateral and Oblique Lateral Lumbar Interbody Fusion and Bilateral Pedicle Screw Fixation Feasibility and Perioperative Results (48)	D.J. Blizzard et al.	Spine	2018	44	IV
13	Complications on minimally invasive oblique lumbar interbody fusion at L2-L5 levels: a review of the literature and surgical strategies (49)	J. Quillo-Olvera et al.	Annals of Translational Medicine	2018	42	I
14	Comparative Study of the Difference of Perioperative Complication and Radiologic Results MIS-DLIF (Minimally nvasive Direct Lateral Lumbar Interbody Fusion) Versus MIS-OLIF (Minimally Invasive Oblique Lateral Lumbar Interbody Fusion) (50)	J. Jin et al.	Clinical Spine Surgery	2018	39	III
15	Retroperitoneal oblique corridor to the L2-S1 intervertebral discs: an MRI study (22)	D.M. Molinares et al.	Journal of Neurosurgery-Spine	2016	39	IV
16	Clinical and Radiologic Outcomes of Direct Versus Indirect Decompression with Lumbar Interbody Fusion: A Matched-Pair Comparison Analysis (51)	G.X. Lin et al.	World Neurosurgery	2018	38	III
17	Outcomes of oblique lateral interbody fusion for degenerative lumbar disease in patients under or over 65 years of age (52)	C. Jin et al.	Journal of Orthopaedic Surgery and Research	2018	30	IV
18	Comparison of Biomechanical Performance Among Posterolateral Fusion and Transforaminal, Extreme, and Oblique Lumbar Interbody Fusion: A Finite Element Analysis (53)	T. Lu et al.	World Neurosurgery	2019	27	V
19	Stereotactic navigation for the prepsoas oblique lateral lumbar interbody fusion: technical note and case series (40)	A.M. DiGiorgio et al.	Neurosurgical Focus	2017	27	IV
20	Comparing stand-alone oblique lumbar interbody fusion with posterior lumbar interbody fusion for revision of rostral adjacent segment disease A STROBE-compliant study (54)	G. Zhu et al.	Medicine	2018	25	IV
21	Lower Lumbar Segmental Arteries Can Intersect Over the Intervertebral Disc in the Oblique Lateral Interbody Fusion Approach with a Risk for Arterial Injury: Radiological Analysis of Lumbar Segmental Arteries by Using Magnetic Resonance Imaging (55)	S. Orita et al.	Spine	2017	25	IV
22	Lateral and Oblique Lumbar Interbody Fusion-Current Concepts and a Review of Recent Literature (56)	R. Hah et al.	Current Reviews in Musculoskeletal Medicine	2019	23	I
23	Comparison of pure lateral and oblique lateral inter-body fusion for treatment of lumbar degenerative disk disease: a multicentric cohort study (57)	M. Miscusi et al.	European Spine Journal	2018	23	IV
24	Preoperative evaluation of left common iliac vein in oblique lateral interbody fusion at L5-S1 (58)	N.S. Chung et al.	European Spine Journal	2017	23	IV
25	Development and Application of Oblique Lumbar Interbody Fusion (59)	R. Li et al.	Orthopaedic Surgery	2020	21	I
26	Oblique retroperitoneal approach for lumbar interbody fusion from L1 to S1 in adult spinal deformity (60)	K.T. Kim et al.	Neurosurgical Review	2018	21	IV
27	Biomechanical Evaluation of Transforaminal Lumbar Interbody Fusion and Oblique Lumbar Interbody Fusion on the Adjacent Segment: A Finite Element Analysis (61)	B. Wang et al.	World Neurosurgery	2019	20	V
28	Comparison Perioperative Factors During Minimally Invasive Pre-Psoas Lateral Interbody Fusion of the Lumbar Spine Using Either Navigation or Conventional Fluoroscopy (62)	Y.H. Zhang et al.	Global Spine Journal	2017	20	III
29	Mini-open oblique lumbar interbody fusion (OLIF) approach for multi-level discectomy and fusion involving L5-S1: Preliminary experience (63)	F. Zairi et al.	Orthopaedics / Traumatology-Surgery / Research	2017	18	IV
30	Imaging Anatomical Research on the Operative Windows of Oblique Lumbar Interbody Fusion (64)	L. Liu et al.	PloS One	2016	18	IV
31	Evolution of Minimally Invasive Lumbar Spine Surgery (2)	A.A. Momin et al.	World Neurosurgery	2020	17	V
32	Lumbar Interbody Fusions for Degenerative Spondylolisthesis: Review of Techniques, Indications, and Outcomes (65)	W.R. Spiker et al.	Global Spine Journal	2019	17	V
33	Lumbar interbody fusion: recent advances in surgical techniques and bone healing strategies (20)	B. Meng et al.	European Spine Journal	2021	16	V
34	Complications Associated with Minimally Invasive Anterior to the Psoas (ATP) Fusion of the Lumbosacral Spine (66)	T. Tannoury et al.	Spine	2019	16	IV
35	Radiographic and Clinical Outcomes of Oblique Lateral Interbody Fusion Versus Minimally Invasive Transforaminal Lumbar Interbody Fusion for Degenerative Lumbar Disease (67)	H.M. Li et al.	World Neurosurgery	2019	16	III
36	Clinical and radiological outcomes of spinal endoscopic discectomy-assisted oblique lumbar interbody fusion: preliminary results (36)	D.H. Heo et al.	Neurosurgical Focus	2017	16	IV
37	Does right lateral decubitus position change retroperitoneal oblique corridor? A radiographic evaluation from L1 to L5 (68)	F. Zhang et al.	European Spine Journal	2017	15	IV
38	Standalone oblique lateral interbody fusion vs. combined with percutaneous pedicle screw in spondylolisthesis (69)	W. He et al.	BMC Musculoskeletal Disorders	2020	14	III
39	Anterior lumbar fusion techniques: ALIF, OLIF, DLIF, LLIF, IXLIF (70)	J. Allain et al.	Orthopaedics / Traumatology-Surgery / Research	2020	14	V
40	Correction of marked sagittal deformity with circumferential minimally invasive surgery using oblique lateral interbody fusion in adult spinal deformity (71)	S.W. Park et al.	Journal of Orthopaedic Surgery and Research	2020	14	IV
41	Minimally Invasive Oblique Lumbar Interbody Fusion with Spinal Endoscope Assistance: Technical Note (72)	D.H. Heo et al.	World Neurosurgery	2017	13	IV
42	Learning Curve of Minimally Invasive Surgery Oblique Lumbar Interbody Fusion for Degenerative Lumbar Diseases (73)	C. Liu et al.	World Neurosurgery	2018	13	IV
43	Modic Changes (MCs) Associated with Endplate Sclerosis Can Prevent Cage Subsidence in Oblique Lumbar Interbody Fusion (OLIF) Stand -Alone (74)	J. Liu et al.	World Neurosurgery	2020	12	IV
44	Minimally Invasive Oblique Lateral Lumbar Interbody Fusion Combined with Anterolateral Screw Fixation for Lumbar Degenerative Disc Disease (75)	T. Xie et al.	World Neurosurgery	2020	12	IV
45	Robot-assisted Percutaneous Transfacet Screw Fixation Supplementing Oblique Lateral Interbody Fusion Procedure: Accuracy and Safety Evaluation of This Novel Minimally Invasive Technique (76)	J. Wu et al.	Orthopaedic Surgery	2019	12	IV
46	Stability Evaluation of Oblique Lumbar Interbody Fusion Constructs with Various Fixation Options: A Finite Element Analysis Based on Three -Dimensional Scanning Models (77)	H. Guo et al.	World Neurosurgery	2020	11	V
47	Minimally invasive surgery for degenerative spondylolisthesis: transforaminal or oblique lumbar interbody fusion (78)	S. Sheng et al.	Journal of Comparative Effectiveness Research	2020	11	III
48	Clinical Effects of Oblique Lateral Interbody Fusion by Conventional Open versus Percutaneous Robot-Assisted Minimally Invasive Pedicle Screw Placement in Elderly Patients (79)	S. Feng et al.	Orthopaedic Surgery	2020	11	III
49	Differences in radiographic and clinical outcomes of oblique lateral interbody fusion and lateral lumbar interbody fusion for degenerative lumbar disease: a meta-analysis (80)	H.M. Li et al.	BMC Musculoskeletal Disorders	2019	11	I
50	Morphometric MRI Imaging Study of the Corridor for the Oblique Lumbar Interbody Fusion Technique at L1-L5 (23)	J.X. Li et al.	World Neurosurgery	2018	10	IV

These articles were further classified according to the study design; 39 were original papers, and 11 were review studies. Furthermore, of these 39 original papers, 29 were research articles, seven were anatomical studies, and the remaining three were biomechanical studies.

**Figure 2:**
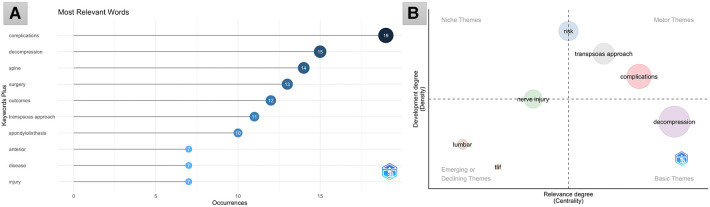
(**A**) The top 10 most cited keywords in the field of pre-psoas oblique lumbar interbody fusion. (**B**) Thematic map. Bottom right is the basic themes, top right is the motor themes, top left is the niche themes, bottom left is the emerging or declining themes.

**Figure 3:**
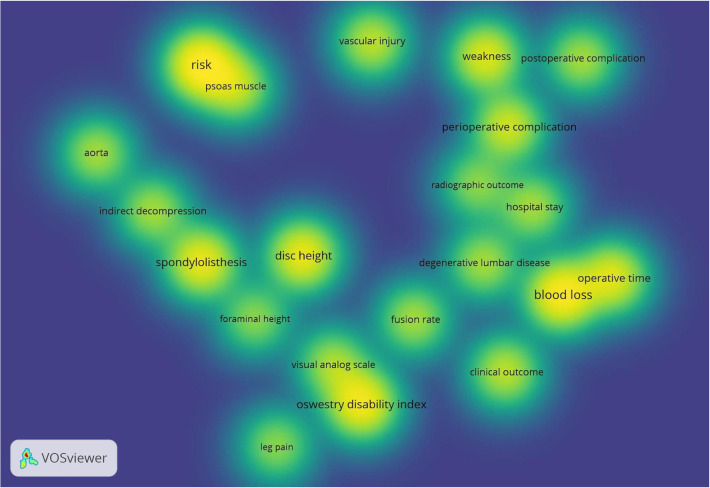
Density visualization map of keywords. The density magnitude depends on the number of elements in the surrounding area and the importance of those elements. The higher the density, the brighter the color; conversely, the lower the density, the lighter the color.

The top 10 most cited keywords were (Figure 2A): “complications,” “decompression,” “spine,” “surgery,” “outcomes,” “transpsoas approach,” “spondylolisthesis,” “anterior,” “disease,” and “injury.” “Complications” is a recent research hotspot and emphasis (Figure 2B). As shown in the keyword density visualization (Figure 3), we could identify perioperative and postoperative complications, as well as clinical and radiological outcomes as the main research hotspots in this field.

## Discussion

The field of OLIF has greatly evolved in recent decades, and this study highlights the 50 most cited articles in this field. Considering the rapid growth of the publications in this day and age, the current screening of the 50 most cited papers in the field of OLIF is valuable for surgeons and researchers to keep them abreast of the most relevant articles, which helps better place the hotspots of this study and helps guide further research efforts in this field.

Among the 50 most cited papers, 29 were clinical research articles, seven were anatomical studies, three were biomechanical studies, and 11 were review articles. Of these 29 clinical studies, six were comparative studies between different techniques, such as two studies comparing OLIF with D/XLIF, three studies comparing OLIF with transforaminal lumbar interbody fusion (TLIF), and one study comparing OLIF with posterior lumbar interbody fusion. The remaining 23 research articles analyzed the clinical outcomes, radiological results, and complications of the OLIF technique. The other 11 review articles were non-meta-analyses in nature, and the authors collected and integrated a large amount of information and addressed it in these reviews, focusing on history, techniques, indications, outcomes, and complications.

### Anatomical and radiological study of the feasibility of the OLIF access

#### Cadaveric study

In one study (19), anatomical data on the OLIF surgical window at L2–S1 were collected from 20 adult cadaveric specimens. The widths of the oblique corridor of the L2–S1 levels were measured in the lateral position, both at rest and with mild distraction of the psoas major muscle. At the L2–L5 level, in the static state, the oblique corridor was the narrowest at 15 mm in L4–L5 and the widest at 19.25 mm in L3–L4; in the mild distraction state, the access corridor increased in all levels, with the highest increase of 59.60% in L2–L3 and the least increase of 43.96% in L3–L4. The L5-S1 disk space is regularly accessible from an oblique angle with gentle iliac vascular retraction.

#### Radiological study

One researcher (22) conducted a magnetic resonance imaging (MRI) study of the width of the L2–S1 OLIF surgical window, which was measured on 100 adult MRI images. The measurements were taken in the static state, and the average width of the surgical window (left side) for each level from L2 to L5 was 16.04 mm for L2–L3, 14.21 mm for L3–L4, and 10.28 mm for L4–L5, and 10 mm for the L5–S1 lateral horizontal width and 10.13 mm for the longitudinal vertical width of the surgical window. Moreover, aortic bifurcation was found to mostly occur in the plane of the L4 vertebral body (43%), followed by the L4–L5 intervertebral disk (11%), the L5 vertebral body (9%), the L3–L4 intervertebral disk (5%), and the L3 vertebral body (2%). Furthermore, a low level of iliac vessel confluence was also found, with 45.9% confluence in the L4 vertebral plane, 19.4% in the L4–L5 intervertebral space, and 34.7% in the L5 vertebral body. In another paper (23), the authors measured the OLIF corridor on the right and left sides in 200 patients. The authors found that the right-side OLIF corridor was much narrower than the left-side one at the same level, indicating that a right-sided approach was less likely to be effective for OLIF.

Anatomical and radiological studies have confirmed the feasibility of the OLIF access; however, a few patients may have a narrow access corridor or anatomical variants; thus, a detailed preoperative examination and careful planning for each patient are required.

### Indications and contraindications

The indications of OLIF have been reported in the literature, which included the following: diskogenic low back pain, lumbar spinal stenosis, lumbar segmental instability, spondylolisthesis, adjacent segmental disease, scoliosis, revision, disk space infection, trauma, and tumors. OLIF can be used for the fusion procedure in the L1–S1 levels; however, this procedure has been available for a short time, and few relevant studies are available, and its indications still need more clinical studies.

There are few reported contraindications to OLIF, which mainly included a history of abdominal surgery, severe obesity, and a narrow OLIF corridor. Furthermore, additional posterior decompression or osteotomy should be combined appropriately in patients with severe nerve root compression, severe spinal stenosis, moderate to severe spinal slippage, bony stenosis of the lateral recess, and moderate to severe rotational spinal deformity.

### Clinical and radiological outcomes

Numerous previous studies and meta-analyses have affirmed OLIF surgical outcomes, such as intraoperative parameters (i.e., operating time, estimated blood loss, and hospital stay), clinical scores (i.e., visual analog scale [VAS] and Oswestry Disability Index [ODI]), and radiographic findings (i.e., restoration of disk height and foraminal height, correction of sagittal and coronal alignment, subsidence, and fusion rate). Woods et al. (24) reported that 137 patients underwent OLIF with an average intraoperative blood loss of 83.2 ml (range, 10–300 ml), and the fusion rate was 97.9% on computed tomography examination after 6 months. A recent meta-analysis included 16 studies (25), which resulted in a mean blood loss of 109.9 ml, a mean operative time of 95.2 min, a mean postoperative hospital stay of 6.3 days, and a postoperative fusion rate of 93%. We retrospectively analyzed the clinical results of 47 patients (62 levels) who underwent OLIF and found significant decreases in the mean VAS scores for back and leg pain, from 6.0 preoperatively to 2.3 postoperatively and from 6.9 preoperatively to 2.2 postoperatively, respectively (26). Simultaneously, the mean ODI decreased from 49.1 preoperatively to 26.5 postoperatively, with a 46.0% improvement (26). Furthermore, according to a recent meta-analysis (27), OLIF effectively corrects sagittal and coronal deformities, in the absence of posterior columnar osteotomy, with a significant difference in VAS and ODI between the preoperative and postoperative periods. Furthermore, the OLIF procedure for treating single-level spinal tuberculosis is more effective than anterior surgery alone, with less trauma and a lower complication rate (28). This may be because the OLIF procedure allows for the direct and complete removal of infectious pathologies from the anterior column and anterior column reconstruction, while using a minimally invasive surgical technique that lowers surgical morbidity.

### Complications

A study (29) reported complications in 155 patients undergoing OLIF, of whom 75 (48.3%) had complications. The most common complications reported were endplate fracture and subsidence and transient low back weakness and thigh numbness at 18.7% and 13.5%, respectively. Other less frequent complications were segmental arterial injury, infection, and revision surgery. Another recent meta-analysis (25) reported intraoperative (1.5%) and postoperative complications (9.9%); the most common complications were transient thigh pain and/or numbness and hip flexion weakness (3.0% and 1.2%, respectively). Although OLIF *via* the prepsoas route is regarded as a generally safe technique, various perioperative and postoperative complications are unavoidable and should require special attention by the surgeon. Possible intraoperative complications of OLIF include the following: vascular injury, nerve injury, sympathetic chain injury, peritoneal and ureteral injury, poor cage position, and endplate violation (30). Possible postoperative complications include the following: buttock and/or thigh pain and/or weakness, superior mesenteric artery syndrome, postoperative ileus, intestinal obstruction, incisional hernia, surgical site or retroperitoneal infection, cage subsidence, and pseudarthrosis (31).

#### Vascular injury (abdominal vessels and segmental arteries)

It is the most serious intraoperative complication of OLIF surgery, and if it occurs, the consequences are unimaginable. Our recommendations are that preoperative imaging determines whether there is an adequate surgical window (OLIF corridor) and that the presence of anatomical variants in the great vessels should be assessed in the surgical area. Moreover, the breakthrough point should not be too far in front of the vertebral body when breaking through the contralateral annule ring. Furthermore, intraoperative hemostasis and neuroprotective measures are required. Regarding the corridor distance, some researchers suggest that this corridor is riskier in patients with a width < 1 cm; others consider that a slight dissection of the psoas major muscle is necessary to obtain a sufficient space for access placement, and overly demanding the width of the corridor is unnecessary.

Segmental arteries are also important vessels susceptible to injury. We recommend that the fixation nail of the OLIF spacer should be inserted as closely as possible to the proximal inferior endplate of the intervertebral space and that the segmental artery alignment area often overlaps with the inferior retraction baffle placement area; thus, placing the stabilizing nail in the superior retraction baffle only is safer. Furthermore, being gentle when installing the spacer and implanting the fixation nail under direct vision are important to ensure the safety of the segmental artery; after the fusion is implanted, the fixation nail and spacer should be withdrawn slowly to confirm that there is no obvious active bleeding.

#### Nerve injury (genitofemoral nerve, sympathetic chain, and lumbar plexus nerve)

Unlike traditional surgery, which requires the decompression of the spinal and nerve root canals and is prone to nerve damage, OLIF requires the stretching of the psoas major muscle, which can easily damage the lumbar plexus nerve, genitofemoral nerve and sympathetic nerve chain, resulting in symptoms, such as radicular pain and abnormal sensation in the lower extremities, numbness and weakness of the psoas major muscle and groin area, and even retrograde ejaculation. On the one hand, postoperative nerve injury symptoms are related to ischemic injury to the lumbar major psoas muscle and lumbar plexus nerves caused by the long duration of surgery and stretching. On the other hand, it can be caused by postoperative hematoma irritation. Generally, no specific treatment is needed, and postoperative recovery can be gradual.

#### Peritoneal and ureteral injury

Peritoneal injury is also one of the complications of OLIF because the peritoneum must be pulled forward when placing the tube. If the peritoneum is not pulled forward enough during the operation and the blunt tissue separation is not sufficient, the peritoneum will be embedded in the gap of the tube and the peritoneum will be torn.

The ureter is located posterior to the peritoneum and descends vertically into the pelvis along the anterior aspect of the medial lumbar major psoas muscle. Its ventral segment is anterior to the vertebral body or anterior to the psoas major muscle, and the ureter can be injured intraoperatively by traction or instrumentation. Ureteral injury can have serious consequences, such as hematuria and urinary extravasation. Therefore, when we establish the channel intraoperatively, we must not operate through the fat; otherwise, the ureter can be easily damaged later in the operation. The extraperitoneal fat must be pushed to the ventral side, and if some of the fat is left under the channel, it will increase the risk of ureteral injury. If ureteral injury occurs, surgical intervention must be performed immediately.

#### Cage subsidence

Mild cage subsidence is a process in which the cage and the upper and lower endplates adhere to each other. When the patient stands up after surgery, the cage is stressed by the endplate and settles to a certain extent so that the cage can make better contact with the upper and lower endplates, and this process results in a partial loss of disk height. Subsidence usually ends when the interface heals and is unlikely to cause serious consequences unless it leads to foraminal narrowing and nerve root compression. According to the previous studies (32–34), elderly, osteoporosis, severe multifidus muscle fatty degeneration, low Hounsfield units, concave endplate morphology, and higher cage height were all risk factors for OLIF subsidence. To avoid cage subsidence, we should choose a fusion device of the appropriate size; avoid intraoperative damage to the bony endplate; choose the appropriate indications; improve osteoporosis; and avoid premature weight-bearing. For patients with severe osteoporosis and significant lumbar instability, a combination of posterior percutaneous arch nail–rod system is required.

#### Nonunion

During OLIF surgery, the imperfect instrumentation of disk tissue removal can easily lead to the incomplete removal of disk tissue. Moreover, osteoporosis can accelerate bone graft resorption, inhibit and reduce new bone formation, and is harmful to bone fusion (20). Furthermore, cage subsidence is also a risk factor for nonunion (35).

### Recent progress

OLIF is an indirect decompression procedure, and to overcome the indirect decompression effect of OLIF, some scholars have attempted to perform disk removal and endplate preparation under spinal endoscopic assistance, and the position of the interbody fusion cage can be assessed under direct vision, which also reduces radiation. Heo et al. (36) published the preliminary results of the OLIF technique with spinal endoscopic assistance (14; 18 segments) and showed that the average time for a single-level procedure is 120 min and that a significant postoperative improvement in preoperative VAS and ODI scores was observed, with the restoration of disk height and foraminal height and an increment of segmental lordosis and whole lumbar lordosis. Another recent article (37) reported the results of their full-endoscopic OLIF, showing significant relief of back and leg pain in all patients (20 patients; 22 levels) and complete interbody fusion in all segments after 1 year.

Recently, intraoperative navigation systems and robotic assistance have been increasingly used in OLIF, and there is also evidence that using these technologies can increase surgical precision and patient outcomes while lowering radiation exposure for surgeons and surgical personnel (38–41).

### Limitations

Citation analysis is a popular bibliometric approach for analyzing scientific publications; however, it has numerous drawbacks. First, we only analyzed papers from journals indexed by the WoS; therefore, some novel papers related to OLIF may have been ignored and excluded from this study. Second, because papers were classified based on the number of citations, certain recent noteworthy papers in the area did not have enough opportunity to be referenced through other writers. As a result, some of the authors' innovative strategies and ideas may be overlooked. Third, only published articles were analyzed in this study. Other studies, such as recommendations, conferences, clinical guidelines, and case reports, may also provide useful insights in this area. Fourth, the self-citation phenomenon is also a factor in analyzing the drawbacks of literature research based on the number of citations. Finally, we included some studies involving few cases (at least 12 cases) in this study. However, these articles are also indispensable, as our goal is to provide a global and exhaustive analytical study of the literature in the field of OLIF.

## Conclusions

This study spotlights the top 50 most cited articles in the OLIF field, which were subjected to a comprehensive bibliometric analysis, including the number of publications per year, number of citations, authors, journals, countries, and research topics. Clinical studies comprised the majority of the studies reviewed, followed by review articles and anatomical studies, and a few were biomechanical studies. Similar to most surgical procedures, the main research topics in the field of OLIF are focused on anatomical morphology, surgical techniques, indications, outcomes, and complications. Research has concentrated on complications, clinical outcomes and radiological outcomes. Complications are a recent research hotspot and focus. Despite the inherent limitations of bibliometric studies based on citation count, with the results of this review, we provide spine surgeons with research priorities and hotspots in the field of OLIF.

## Data Availability

The original contributions presented in the study are included in the article/Supplementary Material, further inquiries can be directed to the corresponding author/s.
